# Ribavirin for Lassa Fever Postexposure Prophylaxis

**DOI:** 10.3201/eid1612.100994

**Published:** 2010-12

**Authors:** Christiane M. Hadi, Augustine Goba, Sheik Humarr Khan, James Bangura, Mbalu Sankoh, Saffa Koroma, Baindu Juana, Alpha Bah, Mamadou Coulibaly, Daniel G. Bausch

**Affiliations:** Author affiliations: Tulane University Health Sciences Center, New Orleans, Louisiana, USA (C.M. Hadi, D.G. Bausch);; Ministry of Health and Sanitation, Kenema, Sierra Leone (A. Goba, S.H. Khan, J. Bangura, M. Sankoh, S. Koroma, B. Juana);; International Center for Research on Tropical Infections, N’Zérékoré, Guinea (A. Bah, M. Coulibaly);; Tulane School of Public Health and Tropical Medicine, New Orleans (D.G. Bausch)

**Keywords:** Lassa fever, arenavirus, ribavirin, postexposure prophylaxis, Sierra Leone, viruses, letter

**To the Editor:** Lassa fever is an acute, viral, hemorrhagic illness endemic to West Africa. Intravenous ribavirin drastically reduces deaths from Lassa fever (*1*). During outbreaks, oral ribavirin is often considered for postexposure prophylaxis (PEP), but no systematically collected data exist for this indication of drug use (*1**–**5*). We therefore conducted a retrospective follow-up study to examine adherence and adverse effects associated with oral ribavirin given as PEP during an outbreak of Lassa fever in Sierra Leone in 2004 (*6*). During this outbreak, family members and some healthcare workers who had direct contact with patients did not use personal protective equipment and were subsequently prescribed oral ribavirin as PEP (200 mg capsules; Schering-Plough Corporation, Kenilworth, NJ, USA).

Approximately 3 months after the possible exposures, we surveyed 23 (92%) of 25 persons known to have been prescribed ribavirin PEP. Respondents were asked about demographics, medical history, details of possible exposure to Lassa virus (LASV), dosage and duration of ribavirin prescribed and taken, and use of concomitant medications. When possible, serum was obtained and tested by ELISA for LASV-specific immunoglobulin (Ig) M and IgG (*7*).

The mean age of the 23 respondents was 38 years (range 23–73 years); 14 (61%) were male, 17 (74%) had been exposed at home (during bathing, cleaning, and feeding of family members with Lassa fever), and 6 (26%) had had in-hospital contact with blood and bodily fluids. No needle-stick injuries were reported.

All respondents had begun taking oral ribavirin within 2 days after exposure. The drug was prescribed at a mean dose of 800 mg 1×/d (most often as 400 mg 2×/d) for 10 days; however, respondents reported actually taking 400–1,200 mg/d. Only 10 (43%) completed the full 10 days of therapy; mean duration of therapy was 8 days (range 1–14 days). No correlation was found between the prescribed daily dose of ribavirin and the likelihood of completing therapy (p = 0.60).

Therapy was completed by 6 (38%) of the 16 (70%) respondents who reported having experienced minor adverse effects and by 4 (57%) of the 7 who reported not having experienced adverse effects (Figure). Many respondents reported having had symptoms even before beginning ribavirin, suggesting at least a partial psychosomatic or other etiology. No correlation was found between likelihood of adverse effects and age (p = 0.18), sex (p = 0.16), or place of exposure (p = 0.63). Six (26%) respondents reported having premorbid health conditions (gastric ulcers, n = 3; gastroesophageal reflux disease, n = 2; hypertension, n = 1), and 15 (65%) took medications in addition to ribavirin during the postexposure period, including paracetamol, folic acid, multivitamins, iron, antacids, antimalarial drugs, antimicrobial drugs, and nonsteroidal anti-inflammatory drugs.

**Figure:**
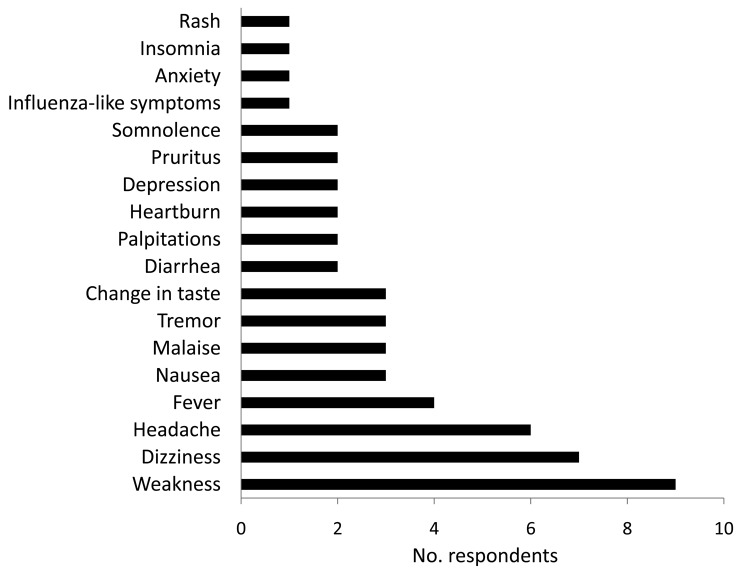
Adverse effects reported by 23 persons who took oral ribavirin prophylactically after potential exposure to Lassa virus, Sierra Leone, 2004.

Minor adverse effects from oral ribavirin PEP, either biologic or psychosomatic, were frequently noted and decreased adherence. Many of the same adverse effects have been reported (*8*). Because interviews in our study were conducted months after medication had been taken, recall bias may have occurred. However, 11 (85%) of the 13 repondents who reported not completing therapy could show the interviewer their leftover ribavirin capsules, thus validating their reports. The observational nature of our study prevented us from establishing a causal association between taking ribavirin and the reported adverse effects. Other factors, especially the anxiety often associated with possible LASV exposure, likely contributed to the noted symptoms.

Although we cannot exclude the possibility of asymptomatic infection, we found no evidence of secondary transmission of LASV among the respondents. One person reported having fever and malaise after exposure, but test results for LASV were negative. Only 8 (35%) persons consented to follow-up laboratory testing, probably because most did not think it was necessary because of lack of symptoms; all 8 were LASV IgM negative. The duration of IgM after LASV infection has not been well characterized, and antibodies could have cleared in the 3 months between exposure and testing (*7*). Another possibility is that swift administration of ribavirin blunted the antibody response. Although not studied in humans, total Ig titers in LASV-infected, ribavirin-treated monkeys eventually reached titers similar to those in untreated monkeys (*9*). Three persons were LASV IgG positive, indicating past exposure. All 3 had other risk factors for infection in addition to their recent exposure, including residence in a Lassa fever– hyperendemic area (all 3) and occupation as healthcare workers (2 of 3).

The limitations inherent in our study are its small sample size and retrospective, uncontrolled design. Considering the relatively low secondary attack rate, the restriction of LASV endemicity to remote areas of West Africa, and the infrequency of high-risk exposures, controlled trials for ribavirin PEP in Lassa fever will probably never be possible. Experiences in the field must therefore be used to inform future decisions with regard to use of ribavirin for this indication. Use of oral ribavirin PEP for Lassa fever is likely to be challenging because of poor adherence and adverse effects.
